# Dynamic cyclin profiles as a tool to segregate the cell cycle

**DOI:** 10.1186/1753-6561-7-S6-P23

**Published:** 2013-12-04

**Authors:** David Garcia Munzer, Margaritis Kostoglou, Michalis C Georgiadis, Efstratios N Pistikopoulos, Athanasios Mantalaris

**Affiliations:** 1 Biological Systems Engineering Laboratory, Centre for Process Systems Engineering, Department of Chemical Engineering, Imperial College London, London, SW7 2AZ, UK; 2 Department of Chemistry, Aristotle University of Thessaloniki, Thessaloniki, 54124 Greece; 3 Department of Chemical Engineering, Aristotle University of Thessaloniki, Thessaloniki, 54124 Greece

## Background and novelty

Mammalian cells growth, productivity and cell death are highly regulated and coordinated processes. The cell cycle is at the centre of cellular control and should play a key role in determining optimization strategies towards improving productivity [1]. Specifically, cell productivity is cell cycle, cell-line and promoter dependant [2]. The cyclins are key regulators that activate their partner cyclin-dependent kinases (CDKs) and target specific proteins driving the cell cycle. To our knowledge, there is no information on cyclin phase-dependent expression profiles of industrial relevant mammalian cell lines. We use the cyclin profiles as a tool to identify and quantify the landmarks of the cell cycle and implement a modelling approach to describe the bioprocess. Hereby, we introduce two possible experimental approaches to obtain such dynamic cyclin profiles.

## Experimental approach

Cyclin expression (cyclin E - G_1 _class and cyclin B - G_2 _class) was studied in GS-NS0 batch cultures by flow cytometry. Two set of experiments were performed: a) culture of cells under perturbed (cell arrest) and unperturbed growth (control run) and b) culture of cells for DNA labelling to perform a proliferation assay as well as a non-exposed cells (control run). The static profiles were obtained by direct cyclin staining and the dynamic profiles were reconstructed by either a) tracking a partially synchronized population or b) combining the timings from proliferation assays with the static profiles.

## Result discussion

Both cyclins showed a clear cell cycle phase-specific pattern (cyclin E was 10% higher at G_1 _and cyclin B was 40% higher at G_2_). These results were consistent among all the different culture conditions and were inferred from the static cyclin profiles. After the arrest release the dynamic cyclin profiles can be directly reconstructed by plotting the relevant cyclin content from the partially synchronized moving population traversing the cycle. An advantage of this approach is a clear view of the cyclin accumulation and transition threshold levels. However, this approach requires testing using different arrest agents, exposure levels and timings, which could have an effect on the cell behaviour.

A second approach included an indirect dynamic cyclin profile reconstruction by combining the acquired proliferation times for different cell cycle phases (e.g. G_1_/G_0_, G_2_/M) with the static cyclin profiles. If the static cyclin profiles are considered as the most representative cyclin values (and near to the transition threshold level), it is possible to reconstruct the dynamic profile by linking the threshold values with the cycling times (from the proliferation assay). The advantage of such approach is the ability to formulate different dynamic cyclin profiles such as constant functions, piece-wise linear functions or more elaborated profiles. However, implementation of such an approach requires the tuning of the proliferation assay and the frequency of sampling since it will affect the quality of the assay.

The two approaches showed comparable results both for the static cyclin profiles (also when compared to the control runs) and the dynamic cyclin profiles.

## Conclusions

The different approaches for deriving the dynamic cyclin profiles provide a versatile experimental toolbox for cell cycle characterization. Cyclins can be used as cell cycle distributed variables and be experimentally validated (quantitatively), avoiding the use of weakly supported variables (e.g. age or volume). The observed patterns and timings provide a blueprint of the cell line's cell cycle, which can be used for cell cycle modelling. The development of these models will aid the systematic study of the cell culture system, the improvement of productivity and product quality.
